# A Review of *Strongyloides* spp. Environmental Sources Worldwide

**DOI:** 10.3390/pathogens8030091

**Published:** 2019-06-27

**Authors:** Mae A. F. White, Harriet Whiley, Kirstin E. Ross

**Affiliations:** Environmental Health, College of Science and Engineering, Flinders University, GPO Box 2100, Adelaide 5001, Australia

**Keywords:** *Strongyloides* spp., *Strongyloides stercoralis*, *Strongyloides fuelleborni*, strongyloidiasis, environmental reservoirs

## Abstract

*Strongyloides* spp. are parasitic nematodes that are transmitted through the environment and are capable of causing disease. These nematodes affect an estimated 3–300 million humans worldwide. Identifying the environmental reservoirs of *Strongyloides* spp. is essential for the development of appropriate control strategies. This systematic literature review examined all published studies that identified *Strongyloides* *stercoralis*, *Strongyloides* *fuelleborni*, *Strongyloides* *fuelleborni* *kellyi*, and *Strongyloides* spp. from an environmental source. Most studies detected the nematode from dog and primate fecal samples. Other environmental sources identified were ruminants, cats, rodents, insects, water, soil, as well as fruit and vegetables. Most studies used microscopy-based identification techniques; however, several employed molecular-based techniques, which have become increasingly popular for the detection of *Strongyloides* spp. A limitation identified was a lack of studies that comprehensively screened all potential environmental samples in a region. Future research should undertake this holistic screening process to identify which environmental reservoirs pose the greatest significance to human health. Potential controls can be identified through the identification of environmental sources. Understanding where *Strongyloides* spp. is commonly found within the environment of endemic areas will inform environmental control strategies to reduce this neglected disease.

## 1. Introduction

Strongyloidiasis is a disease caused by parasitic nematodes of the genus *Strongyloides*. Within this genus, three species, *Strongyloides stercoralis, Strongyloides fuelleborni*, and *Strongyloides fuelleborni kellyi* are known to parasitize humans [1,2]. 

*S. stercoralis*, *S. fuelleborni*, and *S. fuelleborni kellyi* are capable of autoinfecting the host. This occurs after adult female parthenogenic nematodes within the infected human shed eggs. These eggs develop to larvae that are passed within the stool. A certain number burrow through the wall of the large intestine, thereby reinfecting the body. Infected individuals can have a low-level undetected infection for many years [3]. When this auto-infective life cycle becomes uncontrolled in immunocompromised, young, and elderly patients, a disseminated infection can develop. Disseminated infection occurs when the parasite travels throughout the body. This can result in sepsis, bacterial meningitis, or gastrointestinal hemorrhage [4]. The mortality rate from a disseminated infection and its comorbidities is estimated to be 80% [3]. The larvae that are passed within the stool are then capable of completing a free-living cycle, in which they molt twice to develop into filariform larvae. These infective filariform larvae are capable of then reinfecting humans, where they can be involved with the autoinfection cycle again [5].

Both *S. stercoralis* and *S. fuelleborni* are able to complete their life cycle within animals such as canids, primates, and insects. Animal species-specific strains of *S. stercoralis* unable to infect humans have been identified [6]. This ability for the nematode to reproduce within other animals indicates that all infected animals’ feces may pose an infection threat to humans. 

After excretion in the stool, larvae can survive and reproduce within the environment, and environmental sources contaminated with larvae can cause reinfection. Although *Strongyloides* spp. are classified as soil-transmitted helminths, locations that harbor *Strongyloides* spp. within the environment, with the exception of soil, have not been investigated holistically [7,8]. By reviewing and collating all reported environmental sources of *S. stercoralis*, *S. fuelleborni*, and *S. fuelleborni kellyi*, environmental interventions can be implemented. 

We need a better understanding of the environmental sources of *Strongyloides* spp.; resistance to the current anthelminthic drugs has been observed in other *Strongyloides* spp. [9]. Both environmental and clinical control of *Strongyloides* spp. is essential [10]. The aim of this review is to identify all research reporting *S. stercoralis*, *S. fuelleborni*, *S. fuelleborni kellyi,* and *Strongyloides* spp. within environmental sources worldwide. 

## 2. Results

One thousand two hundred and twenty-two papers were retrieved from SCOPUS and Web of Science using the search terms identified as suitable, as seen in Table 1, with 174 articles identified as eligible for inclusion.

*S. stercoralis* was identified in 35% of all studies and *S. fuelleborni* in 10% of all studies; both *S. stercoralis* and *S. fuelleborni* were identified in 1% of all studies. *S. fuelleborni* and *Strongyloides* spp. were identified in 0.5% of studies, and genus-level identification was identififed in 55% of all studies, as seen in Table A1. *S. fuelleborni kellyi* was not identified within any papers. 

The most commonly identified reports of *Strongyloides* spp. were within primates (26% of all published works), and dogs (14% of all published works), as seen in Table A1. Other animals identified as environmental sources included cats, ruminants, rodents, and insects. Water, soil, as well as fruit and vegetables were all also identified as containing *Strongyloides* spp. 

Fifty percent of all studies identifying *Strongyloides* spp. within primate populations identified the larvae to genus level only. *S. fuelleborni* was the next most frequently identified species at 40%. Parasitic infections were identified more frequently in terrestrial primates than arboreal primates [11,12]. Most studies (80%) employed microscopy, as seen in Table A1. Proximity to human populations and increased interaction with human populations was also frequently reported in infected populations [13,14]. Captive primates treated with anthelmintic drugs were also reported as carriers of *Strongyloides* spp. [15]. Sample size ranged from 7 to 3349, and prevalence within primate studies ranged from <1% to 100%, as seen in Table A1. 

Domestic and stray dogs were the second most commonly identified source. Fourteen percent of all studies identifying *Strongyloides* spp. were within dogs, with sample sizes ranging from 35 to 879 and prevalence ranged from <1% to 45%, as seen in Table A1. 

All studies reporting incidences of *Strongyloides* spp. within ruminant farming animals only identified *Strongyloides* to the genus level, as seen in Table A1. 

Studies identifying rodents as a source of *Strongyloides* spp. accounted for 5% of the published works. Studies identified *Rattus rattus*, *Rattus norvegicus*, *Mus musculus*, *Dasyprocta*, and *Hydrochoerus hydrochaeris* as carriers of *Strongyloides* spp. [16,17,18,19,20,21,22]. Sample sizes for rodent-based studies ranged from 10 to 502. The prevalence ranged from 10% to 97%, as seen in Table A1. 

Studies identifying insects within the order Diptera as a source of *Strongyloides* spp. accounted for 2% of the published works, as seen in Table A1. Identified insects within this order included flies of the genus *Musca* spp. and *Lucilia* spp. All studies identifying *Strongyloides* spp. within Diptera identified it from sites within the continent of Africa [23,24,25]. The sample sizes ranged from 5000 to 9950, and prevalence was between <1% and 2%.

Insects within the order Blattodea were identified in 2% of all studies, as seen in Table A1. Identified insects within this order include cockroaches from the genus *Periplaneta* spp. and *Blattella* spp. Four of the five identified studies reported *Strongyloides* spp. within populations of Blattodea in Africa. The remaining study identified *Strongyloides* spp. in Blattodea in Thailand. All studies identified infected insects within housing and food preparation areas [26,27,28,29]. The sample sizes of studies identifying insects within the order Blattodea ranged from 70 to 920, with the prevalence ranging from 1% to 81%. 

Half (50%) of published works identifying parasitic contamination of vegetables and fruits found *S. stercoralis* upon leafy, rough-surfaced vegetables such as lettuces, cabbage, celery, spinach, and carrot [30,31,32,33,34,35,36,37]. The sample sizes for fruit and vegetable-based studies ranged from 36 to 1130, with prevalence ranging from <1% to 46%. 

Countries where *Strongyloides* spp. was identified in soils in public areas included Spain, Iran, Malaysia, Nigeria, Brazil, the Czech Republic, Slovakia, and Romania, as seen in Figure 2 [38,39,40,41,42,43,44,45,46,47]. Geophagy, the purposeful consumption of soils, was also commonly identified as a factor in infection from soil-based sources. 

**Figure 1:**
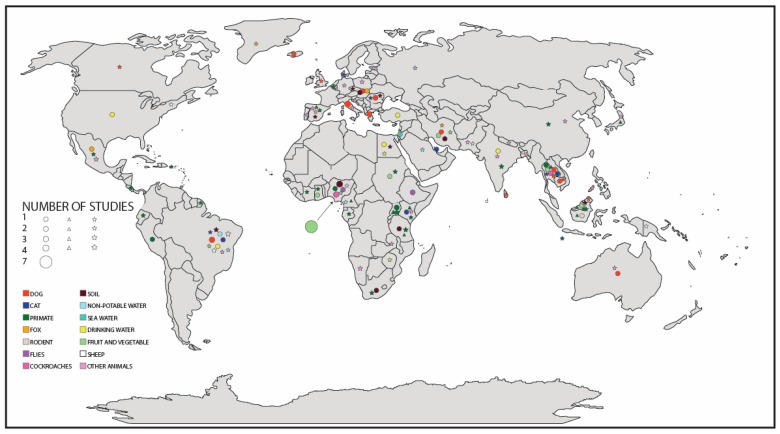
Map displaying the global distribution of all reported environmental cases of *Strongyloides stercoralis*, *Strongyloides fuelleborni*, and *Strongyloides* spp. Where circles are representative of *Strongyloides stercoralis*, diamonds are representative of *Strongyloides fuelleborni*, and stars are representative of *Strongyloides* spp. The size of each shape is mapped to the number of studies published in that country. Location of shapes does not represent exact location of study, but country in which the study was completed. Colored fill of shapes was assigned to a single source and is consistent across all helminth species.

Studies identifying environmental sources of *S. stercoralis*, *S. fuelleborni*, and *Strongyloides* spp. are distributed across the world. Areas with a large amount of research included Europe, Africa, and South East Asia, as seen in Figure 1. Areas lacking research include Oceania, and the Americas, as seen in Figure 1. Many published studies identified *Strongyloides* spp. within temperate regions as opposed to tropical regions, as seen in Figure 1. 

Microscopy was the most commonly used identification technique (90%). However, molecular detection was more common in recent publications. For example, in 2018, 6 of the 14 papers identified employed molecular-based techniques; however, in 2011, 1 of 11 papers published used molecular-based techniques, as seen in Table A1.

## 3. Discussion

### 3.1. Animals 

Primates and domesticated or feral dogs (canids) adapt well to association with human settlements and cohabitation with humans, indicating the potential for transmission to humans. Contamination with feces from domesticated or synanthropic primates and dogs may lead to other environmental sources, such as water and soil, becoming reservoirs of *Strongyloides* spp. capable of causing infection. Most studies found in this review were based on primate and dog investigation, suggesting that these animals preferentially live closely with and benefit from humans. This habitual closeness presents a chance for environmental transmission of *Strongyloides* spp. 

#### 3.1.1. Canids

Studies that report parasites found in canid feces frequently investigate multiple parasites such as *Ancylostoma* spp., *Giardia* spp., and *Strongyloides* spp. These studies have often found low levels of *S. stercoralis* within otherwise highly parasitically infected populations [40,48,49,50,51,52,53,54,55,56]. Infection occurs more frequently in canids when they are living stray. This might be a result of exposure to infective *Strongyloides* spp. larvae occurring more frequently to these dogs than dogs living within homes [57]. Mass drug administration (MDA) to stray dogs has been implemented successfully for the control of rabies; accordingly, it may be an option for the control of *Strongyloides* spp. [10]. Isolated or infrequent anthelmintic treatment increases infection rates and so considered treatment must be implemented [10,58]. Studies identifying canid feces as containing *Strongyloides* spp. commonly also screened the samples for other parasites. Sample sizes ranged from 35 to 3465. The highest prevalence was reported by Beknazarova et al. [59] who screened 35 canine fecal samples from Australia, of which 49% were positive for *Strongyloides* spp. This low sample size with a high positivity rate in comparison with other studies is representative of the inconsistent fecal shedding of *Strongyloides* spp. as well as the endemic location of the study. The lowest prevalence was reported by Ardelean et al. [60] with 1% of 3465 samples positive from dogs within Romania. Strongyloidiasis was observed most commonly in dogs three to six months of age in this study. This variance based on age and study location may be further impacted by the detection method. Ardelean et al. [60] reported high levels of *Ancylostomidae* spp. which is morphologically similar to *Strongyloides* spp., therefore making reliable identification with microscopy alone difficult. 

#### 3.1.2. Primates

Areas sparsely populated by humans increase roaming in primates due to the attractive food sources but offer a low threat from the decreased human numbers. More frequent entry to communities in search of food potentially increases the numbers of *Strongyloides* spp.-infected primate feces within these sparsely populated communities [13,14]. Terrestrial *Papio* primates were likely to excrete *Strongyloides* spp. larvae; however, arboreal *Cercopithecus neglectus* were less likely [11,12]. This may be due to less frequent contact with soil containing *Strongyloides* spp. larvae. The impact of human populations upon forests has led to an increased chance of interaction between humans and potentially infected primates. Hasegawa et al. [61] observed that degraded forest increased the chance of roaming and transfer of parasites. 

Captive primates present an infection risk to handlers because anthelmintic treatment has been observed to not eliminate *Strongyloides* spp. larvae shedding within feces [15]. This may be due to the introduction of new individuals to groups, a phenomenon also observed within wild individuals [15]. This indicates the value in introducing physical environmental controls beyond anthelmintic drugs, especially in communities exposed to roaming wild primates. 

Tourist sanctuaries provide an ideal environment for contact between primates and humans. Environmental controls such as fecal contamination removal can decrease helminthic infection in both primates and humans without interfering with natural behaviors [62]. *Strongyloides* spp. is unable to transfer either from animal to human or from human to human directly [63]. This further supports the importance of clearing feces because contact with the animals does not cause infection; however, contact with fecal matter can cause infection. Larger groups, such as those within tourist sanctuaries, are generally associated with higher parasitic species richness. Some variation of infection can be expected based on food availability and stress levels [64]. 

Studies of primates had sample sizes ranging from 7 to 3349, with prevalence also ranging from <1% to 100%. Prevalence within primate populations was reported to be higher than in canine populations. Hasegawa et al. [61] reported 100% prevalence within seven gorilla and chimpanzees from Uganda; whereas Li et al. [65] screened 3349 fecal samples and identified a prevalence rate of 6%. This variation in prevalence may be due to the inconsistent shedding of *Strongyloides* spp. larvae. Li et al. [65] employed microscopy whereas Hasegawa et al. [61] employed molecular techniques, which may account for differentiation in prevalence. 

#### 3.1.3. Ruminants

*Strongyloides* spp. has also been found within the feces of ruminants used in western farming settings including pigs, sheep, and cattle. All studies identifying *Strongyloides* spp. within farm-associated ruminants only identified the parasite to the species level [66,67]. These may have been genus specific, such as the pork threadworm, *Strongyloides ransomi,* or the more general *Strongyloides papillosis*. All studies used microscopy, a technique that can have low success in identifying *Strongyloides* spp. to species level. These recorded observations indicate the potential for infected ruminant feces to provide an environmental source of *Strongyloides* spp. 

#### 3.1.4. Rodents

Rodents are known to carry a range of communicable diseases. *Strongyloides* spp. has been found in several rodent species, including common house rats, and non-synanthropic rodents such as *Hydrochoerus hydrochaeris*. *S. stercoralis* has been identified in house rat feces in East Java, Indonesia, using microscopy [20]. The area in which *S. stercoralis* was identified in house rat feces is an area with poor sanitation and hygiene. People reported a large house rat population within these areas [20]. In such cases, where a zoonotic pathogen is identified, control of the offending carrier can be employed. The sample sizes of rodents were low in comparison with other sources, with the highest sample size being 502 [19]. The highest prevalence was within a population of *Rattus norvegicus* within a Brazilian slum [17]. The rodents sampled within this study had particularly high levels of infection with helminths; in all except five, helminths were present within their feces [17]. This prevalence of 97% from a sample size of 299 is representative of animals living within an area highly contaminated by human waste.

#### 3.1.5. Insects

Increasing urbanization has allowed for synanthropic dependence to increase within insect populations. Densely urbanizing areas lead to an increase in available food for insects, and areas with poor sanitation and hygiene practices attract disease-carrying insects such as those within the order Blattodea (cockroaches) and Diptera (flies). Filth flies present a source of helminth transmission. Their preference for consuming wet, rotting substances indicates a high probability for the consumption and carriage of *Strongyloides* spp. Carriage of *Strongyloides* spp. has been observed on the external body of flies despite frequent preening and cleaning [68]. Fetene and Worku [23] identified *S. stercoralis* within *Chrysomya rufifacies*, *Musca sorbens*, and *Lucilia cuprina*. *C. rufifacies* were identified largely within butcheries and defecating grounds; *M. sorbens* was found more frequently within the market collection sites. Furthermore, *Musca domestica*, a species always found in association with humans, has been observed to carry *Strongyloides* spp. [24]. The presence of these flies within human food areas presents a potential transmission route for *Strongyloides* spp. larvae. Prevalence was higher in the internal structures of flies than on the external surface of flies [25]. This observation is further supported by the preference of these flies for consumption of wet substances. Insects within the order Blattodea, commonly known as cockroaches, also present a transmission source. Parasite prevalence has been found to be associated with housing type. Low-cost housing with pit latrines as well as housing in close proximity to dumpsites was reported to contain higher levels of carrier cockroaches [26,28,29]. Through the introduction of environmental controls such as fly screens or nets, movement of carrier insects can be decreased [27]. Sample sizes for both orders were high. Studies found low prevalence with the exception of Morenikeji et al. [28] who reported 81% in 70 cockroaches. 

### 3.2. Water 

Contamination of water is also a potential source of helminth transferal. Pollution of water sources with human and agricultural waste can render water sources unsuitable for use as drinking and irrigation water. In areas where water access is limited, contaminated water may be employed for these uses [69]. Waste stabilization ponds, chlorination, or activated sludge treatment systems may be suitable approaches for reducing helminth levels; however, many studies monitoring wastewater treatment methods have provided contradictory results [70,71,72,73]. Some studies identified standard treatment techniques as adequate for removing larvae; however, others did not. Frequent monitoring of treated waste water is important because treated water has been identified as containing higher than acceptable levels of helminths including *Strongyloides* spp. [74]. To date, studies have focused on the helminth burden of treated water instead of comparing treated with untreated levels. Through focusing on untreated and treated waters from the same area, reduction in burden levels of treated water may be better understood. 

Untreated water used for drinking can contain *Strongyloides* spp., particularly when water is sourced from storm water or collected rainwater [75]. According to one study, when water runoff moves into drinking water sources such as rivers, it can carry *Strongyloides* spp. larvae with it [75]. Bore and ground-water contamination can also occur and has been identified [76,77]. Jonnalagadda and Bhat [77] found that improper washing of vessels used to collect and store water can lead to helminth contamination. Implementation of appropriate washing and sanitation education in areas with high contamination risk may decrease incidences of infection. 

Prevalence of *Strongyloides* spp. within non-potable water was higher than in potable water; this was expected because most sources of non-potable water were collected from wastewater treatment facilities [71]. The prevalence of *Strongyloides* spp. within non-treated wastewater was between 40–100%. Treated waste water intended for use on crops for human consumption had a much lower prevalence (2%); however, it was still observed to be present, supporting the importance of monitoring water intended for reuse [70].

### 3.3. Fruit and Vegetables 

The rough nature of green, leafy vegetables surfaces means that adhesion of parasitic larvae and eggs occurs easily when these vegetables are either washed with contaminated water or come into contact with contaminated human fecal-based fertilizers (i.e., night soil) [30]. Studies identified *S. stercoralis* contamination most frequently within leafy, rough-surfaced vegetables such as lettuces, cabbage, celery, spinach, and carrot [30,31,32,33,34,35,36,37]. This correlation may be due to these vegetables growing close to or in the ground, which may lead to increased contamination from fertilizers [78,79]. Market vendors commonly wash vegetables prior to purchase, and consumption of raw vegetables such as salad leaves is frequently noted [31,79,80]. There is an increasing focus on the study of vegetables, washing water, and farm soil to determine where in the food chain parasites are being introduced [33].

Prevalence of *Strongyloides* spp. within fruit and vegetable samples was generally low, ranging from <1% to 46%. Ogbolu et al. [81] found *S. stercoralis* in 46% of fresh vegetables sold at open markets in Nigeria. The application of night soils and untreated wastewater is common within low-income nations may have led to the high level of prevalence [81]. Lower prevalence was also reported within Nigeria, between <1% and 19%. This variation may be due to differences in handling of samples, treatment during farming, and cross contamination [30,31].

### 3.4. Soil 

Increasing urbanization has led to an ever-increasing amount of waste. Modern waste includes not only fecal waste but waste produced in the form of rubbish. Dumpsites and landfills are commonly employed to deal with this large amount of waste; *Strongyloides* spp. contamination can occur throughout the nearby environments. Dumps and landfills pose a transmission risk due to the ability of *Strongyloides* spp. larvae to survive effectively in the soil [82]. 

Contamination of soils with animal feces within public recreation areas also presents a transmission source. High levels of soil-transmitted helminths were reported in public area soils such as parks in Spain, Iran, Malaysia, Nigeria, Brazil, the Czech Republic, Slovakia, and Romania [38,39,40,41,42,43,44,45,46,47]. 

The texture and chemistry of soil also plays a role in the prevalence of *Strongyloides* spp. larvae. Moisture levels in soils increases the incidence of rhabditiform larvae developing into filariform larvae [83]. This requirement for moisture is supported by the findings of a study of helminth larvae during the wet season, which found that no larvae were located during the dry season despite contamination [39]. High sand and silt content soils favor the survival of *Strongyloides* spp. and other helminth larvae. This is due to the high porosity of these soils, which allows larvae to move effectively through the soils towards sources of nutrition and moisture [84]. 

*Strongyloides* spp. is transmitted from soil-based sources to humans through skin-to-soil contact; however, the behavior of purposefully ingesting soil known as geophagy can also lead to soil-based infections. Geophagy is culturally accepted and common in sub-Saharan Africa. This behavior is common in pregnant women; *S. stercoralis* infections have been observed, along with other soil-transmitted helminth infections, in these women [85,86]. Geophagy may be undertaken as a method for diet supplementation in low-income areas. Notably, these areas are more likely to have helminth-contaminated soil, which leads to an increased chance of infection. 

Prevalence of *Strongyloides* spp. within soil was between 1% and 20%. Ivoke et al. [85] screened 797 pregnant women for parasitic infections related to geophagy. The prevalence of infection was 1% within this cohort. This low prevalence is likely due to picking soils specifically for consumption. Higher prevalence was observed in soils sampled in soil directly from areas densely populated with poor health infrastructure [41].

### 3.5. PCR and Microscopy

Identification of *Strongyloides* spp. nematodes can be undertaken using several methods. These fall into either techniques involving the identification of *Strongyloides* spp. larvae using a microscope or molecular-based techniques. Microscopy presents several problems but it accounts for most larval identification-based techniques (90%) as presented in this literature review. This study did not exclude papers based on year; accordingly, this overrepresentation of microscopy is likely a result of the recent introduction of polymerase chain reaction (PCR) techniques. Recent papers have increasingly employed PCR as accessibility to the equipment increases. In 2018, 5 of the 14 papers published employed molecular-based techniques; in contrast, in 2011, only 1 of 11 studies published employed molecular-based techniques, as seen in Table A1. This increasing use of molecular-based techniques is expected, and its use will allow for more accurate identification of species of *Strongyloides* spp. The strengths of microscopy include that it can be employed within the field or where resources are limited; however, microscopy alone cannot reliably differentiate *S. stercoralis* from *S. fuelleborni*. The reliance on microscopy-based techniques is hazardous because both species are morphologically similar [34]. Molecular techniques allow for the accurate identification of *Strongyloides* spp. to the species level; however, set up of molecular protocols can be expensive. Each technique has strengths and weaknesses; when looking at all published works, a consideration of the identification techniques allows for more accurate assessment of reports. 

### 3.6. Global Distribution

Globally, published research has mainly focused in countries within Africa, Europe, and South East Asia. Commonly, *Strongyloides* spp. is reported as a tropical disease; however, *Strongyloides* spp. was often also reported in temperate regions such as Europe, as seen in Figure 1 [83,87]. This may be due to a lack of resources within low-income nations, leading to an overrepresentation of the generally higher income countries within Europe. Australia and the Americas both lacked studies looking at the environmental sources of *Strongyloides* spp. (Figure 1). This indicates a need for more research into the environmental transmission of *S. stercoralis*, *S. fuelleborni*, and *Strongyloides* spp. within these areas. 

## 4. Materials and Methods 

This systematic literature review is based on an adapted version of the PRISMA statement. This tool allows for the transparent and reliable reporting of evidence. A systematic search of the databases Scopus and Web of Science was undertaken, and all articles published prior to 2019 were included. Key words used in searches included *Strongyloides* spp., strongyloidiasis, tap water, soil, insect, zoonotic, and waste, as seen in Table 1. A search strategy was developed to ensure a transparent and complete literature review of all identified environmental sources of *Strongyloides* spp. was completed. This strategy is as follows; All non-English documents were excluded from the search. 

**Figure 2:**
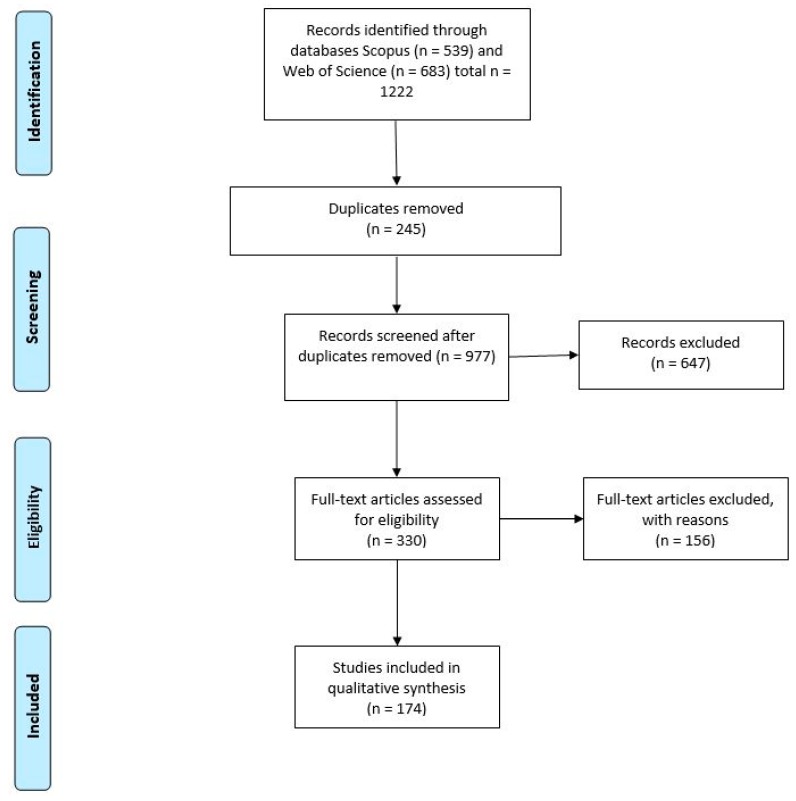
Flow diagram representing the search strategies used (based on the PRISMA statement reporting guidelines for systematic literature reviews) showing an overview of the retrieved articles and the total articles identified as eligible.

To be included, published data must have reported *Strongyloides* spp. in one of the three spp. capable of human infection or to the genus level because these studies cannot be excluded as identifying disease-causing *Strongyloides* spp. The document must have reported this presence within an environmental source. Documents were excluded if they were reviews, reports of humans with *Strongyloides* spp. infection with no mention of contributing environmental source, or lab-based studies, as seen in Figure 2. 

First, all titles and abstracts of all papers were manually reviewed to ensure the papers met inclusion criteria. If it was unclear from titles and abstracts if papers met the criteria, they were included for full text review. Papers that were unclear were included. Papers were then read as full text and compared against inclusion and exclusion criteria. Articles that met these criteria were included in the study. All papers included in the study had key points extracted and recorded including the environmental source reported, species of *Strongyloides* spp. observed, detection method used, and the country from which the sample was taken.

## 5. Conclusions

Although *Strongyloides* spp. is considered a soil-transmitted helminth, there are several environmental sources that can potentially provide a route of transmission of the disease. Understanding the potential sources, combined with the adoption of environmental controls for *Strongyloides* spp. is likely to decrease transmission and therefore infections. Animals such as dogs, primates, and insects, as well as soil, water, and fruit and vegetables have all been reported to contain *Strongyloides* spp. larvae, capable of perpetuating infection within humans who have come into contact. Future research is needed to undertake a holistic screening of all environmental sources within endemic areas to identify those which pose the greatest significance to human health. By understanding the established and recorded environmental reservoirs of *S. stercoralis*, *S. fuelleborni*, and *S. fuelleborni kellyi*, better environmental controls can be implemented. 

## Figures and Tables

**Table 1:** Complete search strategy and all key words used to identify relevant literature.

Search Terms Employed to Identify Relevant Literature
*Strongyloides* OR Strongyloidiasis OR “*Strongyloides stercoralis*” OR “*S. stercoralis*” OR “*Strongyloides fuelleborni*” OR “*S. fulleborni*” OR “*Strongyloides fulleborni kellyi*” OR “*S. fulleborni kellyi*”
AND
“Tap Water” OR “Potable water” OR Water OR Soil OR Dirt OR sediment OR synanthropic OR “synanthropic insect” OR Insect OR “*Musca domestica*” OR flies OR “*Musca vetustissima*” OR *Sarcophagidae* OR “*Chrysomya megacephala*” OR “*Musca sorbens*” OR “*Lucilia cuprina*” OR “*Calliphora vicina*” OR “*Blattella germanica*” OR “*Periplaneta Americana*” OR Cockroach OR dog OR “Canis lupis” OR zoonotic OR Monkey OR “septic tank” OR waste OR wastewater OR rubbish OR trash OR environment

## Appendix A

**Table A1:** Summary of all reports, and studies identifying *S. stercoralis, S. fuelleborni, S. fuelleborni kellyi*, and *Strongyloides* spp. within environmental sources worldwide.

Species Parasite	Prevalence	Sample Size	Detection Method	Country	Reference	Source
*Strongyloides stercoralis*	1%	3465	Microscopy	Romania	(Ardelean et al., 2005) [60]	Dog
*Strongyloides stercoralis*	49%	35	Molecular	Australia	(Beknazarova et al., 2017) [59]	Dog
*Strongyloides stercoralis*	<1%	3208	Microscopy	Iceland	(Eydal and Skirnisson 2016) [88]	Dog
*Strongyloides stercoralis*	<1%	215	Microscopy	Brazil	(Ferreira et al., 2006) [89]	Dog
*Strongyloides* spp.	<1%	457	Microscopy	Canada	(Gaunt and Carr 2011) [90]	Dog
*Strongyloides stercoralis*	<1%	181	Microscopy	Brazil	(Goncalves et al., 2007) [91]	Dog
*Strongyloides stercoralis*	87%	88	Molecular	Cambodia	(Jaleta et al., 2017) [6]	Dog
*Strongyloides stercoralis*	<1%	879	Microscopy	Greece	(Kostopoulou et al., 2017) [48]	Dog
*Strongyloides stercoralis*	<1%	189	Microscopy	Thailand	(Leelayoova et al., 2009) [49]	Dog
*Strongyloides stercoralis*	45%	171	Microscopy	Brazil	(Martins et al., 2012) [58]	Dog
*Strongyloides stercoralis*	4%	52	Microscopy	Romania	(Mircean et al., 2012) [92]	Dog
*Strongyloides* spp.	5%	175	Microscopy	Malaysia	(Noor Azian et al., 2008) [40]	Dog
*Strongyloides stercoralis*	2%	281	Microscopy	Greece	(Papazahariadouet al., 2007) [50]	Dog
*Strongyloides stercoralis*	2%	272	Microscopy	Italy	(Paradies et al., 2017) [93]	Dog
*Strongyloides* spp.	11%	90	Microscopy	Sri Lanka	(Perera et al., 2013) [57]	Dog
*Strongyloides stercoralis*	6%	174	Microscopy	Iran	(Razmi et al., 2009) [52]	Dog
*Strongyloides stercoralis*	<1%	239	Microscopy	Italy	(Riggio et al., 2013) [53]	Dog
*Strongyloides stercoralis*	<1%	639	Microscopy	Italy	(Sauda et al., 2018) [55]	Dog
*Strongyloides* spp.	15%	94	Microscopy	Cambodia	(Schär et al.,2014) [87]	Dog
*Strongyloides stercoralis*	10%	60	Microscopy	Slovakia	(Štrkolcová et al., 2017) [45]	Dog
*Strongyloides* spp.	2%	171	Microscopy	England	(Wright et al., 2016) [56]	Dog
*Strongyloides stercoralis*	<1%	463	Microscopy	Italy	(Zanzani et al., 2014) [94]	Dog
*Strongyloides* spp.	2%	197	Microscopy	Thailand	(Pumidonming et al., 2016) [51]	Dog
*Strongyloides stercoralis*	18%	824	Microscopy	Qatar	(Abu-Madi et al., 2007) [95]	Cat
*Strongyloides* spp.	47%	28	Microscopy	Christmas Island	(Adams et al., 2008) [96]	Cat
*Strongyloides* spp.	54%	37	Microscopy	Brazil	(Lima et al., 2017) [97]	Cat
*Strongyloides* spp.	3%	414	Microscopy	Romania	(Mircean et al., 2010) [98]	Cat
*Strongyloides stercoralis*	14%	173	Microscopy	Brazil	(Monteiro et al., 2016) [99]	Cat
*Strongyloides stercoralis*	44%	103	Microscopy	Kenya	(Njuguna et al., 2017) [100]	Cat
*Strongyloides* spp.	<1%	300	Microscopy	Thailand	(Rojekittikhun et al., 2014) [101]	Cat
*Strongyloides stercoralis*	21%	38	Microscopy	Thailand	(Sedionoto and Anamnart 2018) [102]	Cat
*Strongyloides* spp.	99 1.0%	99	Microscopy	Denmark	(Takeuchi-Storm et al., 2015) [103]	Cat
*Strongyloides fuelleborni*	UNK	UNK	Molecular and Microscopy	Japan	(Arizono et al., 2012) [13]	Primate
*Strongyloides* spp.	41 44%	41	Microscopy	Uganda	(Bezjian et al., 2008) [11]	Primate
*Strongyloides* spp.	37%	24	Microscopy	French Guiana	(De Thoisy et al., 2001) [14]	Primate
*Strongyloides* spp.	21%	125	Microscopy	India	(Ekanayake et al., 2006) [104]	Primate
*Strongyloides fuelleborni*	28%	293	Microscopy	Uganda	(Gillespie et al., 2004) [105]	Primate
*Strongyloides fuelleborni and Strongyloides stercoralis*	<1% *S. stercoralis*, 4% *S. fuelleborni*	2103	Microscopy	Uganda	(Gillespie et al., 2005) [106]	Primate
*Strongyloides fuelleborni*	84%	153	Microscopy	Tanzania	(Gillespie et al., 2010) [107]	Primate
*Strongyloides fuelleborni and Strongyloides* spp.	11% *S. fulleborni*, 15% *S*. spp	27	Microscopy	Spain	(Gomez et al., 1996) [15]	Primate
*Strongyloides fuelleborni*	23%	401	Microscopy	Japan	(Gotoh 2000) [108]	Primate
*Strongyloides fuelleborni and Strongyloides stercoralis*	100%	7	Molecular	Uganda	(Hasegawa et al., 2016) [61]	Primate
*Strongyloides* spp.	88%	96	Microscopy	Ecuador	(Helenbrook et al., 2015) [64]	Primate
*Strongyloides* spp.	4%	238	Microscopy	Uganda	(Hodder and Chapman 2012) [109]	Primate
*Strongyloides* spp.	7%	40	Microscopy	Kenya	(Karere and Munene 2002) [12]	Primate
*Strongyloides* spp.	41%	624	Microscopy	Borneo	(Klaus et al., 2018) [62]	Primate
*Strongyloides fuelleborni*	32%	652	Microscopy	Borneo	(Klaus et al., 2017) [110]	Primate
*Strongyloides fuelleborni*	57%	141	Microscopy	Puerto Rico	(Knezevich et al., 1998) [111]	Primate
*Strongyloides* spp.	43%	686	Microscopy	Tanzania	(Kooriyama et al., 2012) [112]	Primate
*Strongyloides* spp.	74%	3142	Microscopy	Côte d’Ivoire	(Kouassi et al., 2015 [113]	Primate
*Strongyloides* spp.	13%	366	Microscopy	India	(Kumar et al., 2018) [114]	Primate
*Strongyloides fuelleborni*	44% *S. fuelleborni*, 4% *S*. spp.	25	Microscopy	Malaysia	(Kuze et al., 2010) [115]	Primate
*Strongyloides fuelleborni*	95%	20	Molecular and Microscopy	Indonesia	(Labes et al., 2011) [116]	Primate
*Strongyloides* spp.	37%	59	Microscopy	Ethiopia	(Legesse and Erko 2004) [117]	Primate
*Strongyloides* spp.	5%	222	Microscopy	Belgium	(Levecke et al., 2007) [118]	Primate
*Strongyloides* spp.	6%	3349	microscopy	China	(Li et al., 2017) [65]	Primate
*Strongyloides stercoralis*	6%	46	Microscopy	Nigeria	(Mafuyai et al., 2013) [119]	Primate
*Strongyloides* spp.	50%	134	Microscopy	Costa Rica	(Maldonado-Lopez et al., 2014) [120]	Primate
*Strongyloides* spp.	77%	78	Microscopy	Ecuador	(Martin-Solano et al., 2017) [121]	Primate
*Strongyloides* spp.	17%	53	Microscopy	Uganda	(Matsubayashi et al., 1992) [122]	Primate
*Strongyloides fuelleborni*	58%	432	Molecular	Uganda	(McLennan et al., 2017) [123]	Primate
*Strongyloides* spp.	84%	121	Microscopy	Uganda	(Muehlenbein et al., 2005) [124]	Primate
*Strongyloides fuelleborni*	45%	315	Microscopy	Kenya	(Munene et al., 1998) [125]	Primate
*Strongyloides fuelleborni*	21%	297	Microscopy	Kenya	(Muriuki et al., 1998) [126]	Primate
*Strongyloides* spp.	76%	83	Microscopy	Costa Rica	(Parr et al., 2013) [127]	Primate
*Strongyloides* spp.	13%	366	Microscopy	Tanzania	(Petrasova et al., 2010) [128]	Primate
*Strongyloides* spp.	44%	130	Microscopy	Tanzania	(Petrzelkova et al., 2010) [129]	Primate
*Strongyloides stercoralis*	15%	86	Microscopy	Peru	(Phillips et al., 2004) [130]	Primate
*Strongyloides* spp.	43%	47	Microscopy	Gabon	(Pouillevet et al., 2017) [131]	Primate
*Strongyloides fuelleborni*	6%	125	Microscopy	Cameroon	(Pourrut et al., 2011) [132]	Primate
*Strongyloides* spp.	53%	55	Microscopy	Ghana	(Ryan et al., 2012) [133]	Primate
*Strongyloides* spp.	8%	420	Molecular	Mexico	(Solorzano-Garcia and de Leon 2017) [134]	Primate
*Strongyloides fuelleborni*	39%	243	Molecular	Thailand and Laos	(Thanchomnang et al., 2018) [135]	Primate
*Strongyloides* spp.	35%	283	Microscopy	India	(Tiwari et al., 2017) [136]	Primate
*Strongyloides stercoralis*	31%	135	Microscopy	Thailand	(Wenz-Mucke et al., 2013) [137]	Primate
*Strongyloides* spp.	24%	272	Microscopy	South Africa	(Wren et al., 2015) [138]	Primate
*Strongyloides* spp.	24%	332	Microscopy	South Africa	(Wren et al., 2016) [139]	Primate
*Strongyloides fuelleborni and Strongyloides* spp.	UNK	14	Molecular	Malaysian Borneo	(Frias et al., 2018) [140]	Primate
*Strongyloides* spp.	29%	64	Microscopy	Brazil	(De Souza et al., 2012) [66]	Sheep
*Strongyloides* spp.	8%	165	Microscopy	Papua New Guinea	(Koinari et al., 2013) [141]	Sheep
*Strongyloides* spp.	<1%	27	Microscopy	New England	(MacGlaflin et al., 2011) [142]	Sheep
*Strongyloides* spp.	UNK	1798	Microscopy	Brazil	(McManus et al., 2009) [143]	Sheep
*Strongyloides* spp.	2%	275	Microscopy	Greenland	(Andreassen et al., 2017) [144]	Fox
*Strongyloides* spp.	4%	22	Microscopy	Iran	(Dalimi et al., 2006) [145]	Fox
*Strongyloides stercoralis*	16%	249	Microscopy	Mexico	(Hernandez-Camacho et al., 2011) [146]	Fox
*Strongyloides stercoralis*	2%	1198	Microscopy	Slovakia	(Miterpakova et al., 2009) [147]	Fox
*Strongyloides* spp.	65%	60	Microscopy	Pakistan	(Afshan et al., 2013) [16]	Rat
*Strongyloides* spp.	97%	299	Microscopy	Brazil	(Carvalho-Pereira et al., 2018) [17]	Rat
*Strongyloides* spp.	40%	25	Microscopy	Brazil	(Lima et al., 2017) [97]	Rat
*Strongyloides* spp.	13%	76	Microscopy	Bangladesh	(Fuehrer et al., 2012) [18]	Rat
*Strongyloides* spp.	10%	502	Microscopy	Nigeria	(Isaac et al., 2018) [19]	Mouse and rat
*Strongyloides stercoralis*	53%	98	Microscopy	Indonesia	(Prasetyo et al., 2016) [20]	House rat
*Strongyloides* spp.	10%	10	Microscopy	Brazil	(Souza et al., 2015) [21]	Capybaras
*Strongyloides* spp.	10%	31	Microscopy	Brazil	(Gioia-Di Chiacchio et al., 2014) [22]	Capybaras
*Strongyloides stercoralis*	2%	6530	Microscopy	Ethiopia	(Fetene and Worku 2009) [23]	Flies
*Strongyloides stercoralis*	<1%	9950	Microscopy	Ethiopia	(Getachew et al., 2007) [24]	Flies
*Strongyloides stercoralis*	2%	5000	Microscopy	Nigeria	(Umeche 1989b) [25]	Flies
*Strongyloides stercoralis*	12%	749	Microscopy	Nigeria	(Adenusi et al., 2018) [26]	Cockroaches
*Strongyloides stercoralis*	1%	920	Microscopy	Thailand	(Chamavit et al., 2010) [27]	Cockroaches
*Strongyloides stercoralis*	81%	70	Microscopy	Nigeria	(Morenikeji et al., 2016) [28]	Cockroaches
*Strongyloides stercoralis*	UNK	234	Microscopy	Nigeria	(Tatfeng et al., 2005) [29]	Cockroaches
*Strongyloides stercoralis*	2%	125	Microscopy	Nigeria	(Adesewa and Morenikeji, 2017) [82]	Soil
*Strongyloides* spp.	3%	625	Microscopy	Spain	(Dado et al., 2012) [38]	Soil
*Strongyloides* spp.	8%	120	Microscopy	Egypt	(Etewa et al., 2016) [83]	Soil
*Strongyloides stercoralis*	1%	797	Microscopy	Nigeria	(Ivoke et al., 2017) [85]	Geophagy
*Strongyloides stercoralis*	2%	1078	Microscopy	Tanzania	(Kawai et al., 2009) [86]	Geophagy
*Strongyloides stercoralis*	3%	112	Microscopy	Iran	(Motazedian et al., 2006) [39]	Soil
*Strongyloides* spp.	7%	182	Microscopy	Malaysia	(Noor Azian et al., 2008) [40]	Soil
*Strongyloides stercoralis*	20%	102	Microscopy	Nigeria	(Ogbolu et al., 2011) [41]	Soil
*Strongyloides* spp.	5%	2520	Microscopy	Brazil	(Rocha et al., 2011) [42]	Soil
*Strongyloides* spp.	2%	500	Microscopy	Czech Republic	(Valkounova 1982) [43]	Soil
*Strongyloides* spp.	3%	125	Microscopy	Brazil	(Mandarino-Pereira et al., 2010) [44]	Soil
*Strongyloides stercoralis*	14%	14	Microscopy	Slovakia	(Strkolcova et al., 2017) [45]	Soil
*Strongyloides stercoralis*	12%	17	Microscopy	South Africa	(Sumbele et al., 2014) [84]	Soil
*Strongyloides* spp.	4%	45	Microscopy	Romania	(Tudor 2015) [46]	Soil
*Strongyloides stercoralis*	6%	150	Microscopy	Nigeria	(Umeche 1989a) [47]	Soil
*Strongyloides* spp.	6%	16	Microscopy	Brazil	(da Silva et al., 2014) [148]	Soil
*Strongyloides* spp.	UNK	8	Microscopy	Cameroon	(Aghaindum and Landry, 2019) [149]	Non-potable water
*Strongyloides* spp.	40% - 100%	100	Microscopy	Saudi Arabia	(Bolbol 1992) [69]	Non-potable water
*Strongyloides stercoralis*	2%%	UNK	Microscopy	Brazil	(Bastos et al., 2008) [70]	Non-potable water
*Strongyloides* spp.	100%	3	Microscopy	Brazil	(Cutolo et al., 2006) [71]	Non-potable water
*Strongyloides stercoralis*	19%	52	Microscopy	Palestine	(Hilles et al., 2014) [150]	Seawater
*Strongyloides stercoralis*	1%	85	Microscopy	Turkey	(Bakir et al., 2003) [151]	Drinking water
*Strongyloides fuelleborni* and *Strongyloides* spp.	11% *S. fuelleborni*, 15% *S*. spp.	9950	Microscopy	Zimbabwe	(Dalu et al., 2011) [76]	Drinking water
*Strongyloides* spp.	UNK	UNK	Microscopy	Egypt	(El Shazly et al., 2003) [75]	Drinking water
*Strongyloides stercoralis*	7%	80	Microscopy	Egypt	(El-Badry et al., 2018) [152]	Drinking water
*Strongyloides stercoralis*	81%	16	Microscopy	Brazil	(Freitas et al., 2017) [153]	Drinking water
*Strongyloides stercoralis*	51%	232	Microscopy	India	(Jonnalagadda and Bhat 1995) [77]	Drinking water
*Strongyloides stercoralis*	100%	UNK	Microscopy	USA	(Klotz et al., 1992) [154]	Drinking water
*Strongyloides stercoralis*	UNK	UNK	Molecular	Malaysia	(Zeehaida et al., 2011) [155]	Fruit & vegetables
*Strongyloides stercoralis*	<1%	1130	Microscopy	Nigeria	(Adamu et al., 2012) [30]	Fruit & vegetables
*Strongyloides stercoralis*	<1%	960	Microscopy	Nigeria	(Adenusi et al., 2015) [31]	Fruit & vegetables
*Strongyloides stercoralis*	10%	150	Microscopy	Nigeria	(Amaechi et al., 2016) [78]	Fruit & vegetables
*Strongyloides stercoralis*	7%	190	Microscopy	Nigeria	(Amuta et al., 2017) [32]	Fruit & vegetables
*Strongyloides stercoralis*	7%	240	Microscopy	Nigeria	(Dada et al., 2015) [33]	Fruit & vegetables
*Strongyloides* spp.	1%	453	Microscopy	Iran	(Fallah et al., 2016) [34]	Fruit & vegetables
*Strongyloides stercoralis*	36%	360	Microscopy	Ghana	(Kudah et al., 2018) [35]	Fruit & vegetables
*Strongyloides* spp.	13%	108	Microscopy	Brazil	(Luz et al., 2017) [156]	Fruit & vegetables
*Strongyloides* spp.	19%	199	Microscopy	Nigeria	(Maikai et al., 2012) [157]	Fruit & vegetables
*Strongyloides* spp.	11%	36	Microscopy	Malaysia	(Matyusof et al., 2017) [80]	Fruit & vegetables
*Strongyloides stercoralis*	1%	260	Microscopy	Sudan	(Mohamed et al., 2016) [36]	Fruit & vegetables
*Strongyloides stercoralis*	10%	265	Microscopy	Thailand	(Punsawad et al., 2019) [37]	Fruit & vegetables
*Strongyloides stercoralis*	46%	120	Microscopy	Nigeria	(Ogbolu et al., 2009) [81]	Fruit & vegetables
*Strongyloides stercoralis*	14%	140	Microscopy	Iran	(Madadi 2010) [158]	Fruit & vegetables
*Strongyloides stercoralis*	19%	80	Microscopy	Nigeria	(Ohaeri and Unogu 2011) [79]	Fruit & vegetables
*Strongyloides* spp.	7%	15	Microscopy	Zambia	(Berentsen et al., 2012) [159]	Other animals
*Strongyloides* spp.	5%	272	Microscopy	Nepal	(Bista et al., 2017) [160]	Other animals
*Strongyloides* spp.	100%	1	Microscopy	Brazil	(Cardia et al., 2016) [161]	Other animals
*Strongyloides* spp.	4%	432	Microscopy	Spain	(Cordon et al., 2008) [162]	Other animals
*Strongyloides* spp.	40%	52	Microscopy	Russia	(González et al., 2007) [163]	Other animals
*Strongyloides* spp.	2%	956	Microscopy	India	(Gupta et al., 2018) [164]	Other animals
*Strongyloides* spp.	<1%	1005	Microscopy	Germany	(Hallinger et al., 2018) [165]	Other animals
*Strongyloides* spp.	31%	42	Microscopy	Japan	(Hasegawa et al., 2017) [166]	Other animals
*Strongyloides* spp.	<1%	400	Microscopy	Croatia	(Hermosilla et al., 2017) [167]	Other animals
*Strongyloides* spp.	64% - 99%	990	Microscopy	Mexico	(Hu et al., 2018) [168]	Other animals
*Strongyloides* spp.	4%	821	Microscopy	China	(Huang et al., 2014) [169]	Other animals
*Strongyloides* spp.	15%	2280	Microscopy	Pakistan	(Khan et al., 2010) [67]	Other animals
*Strongyloides* spp.	UNK	6	Microscopy	Namibia	(Kumba et al., 2003) [170]	Other animals
*Strongyloides* spp.	36%	58	Microscopy	Poland	(Mizgajska-Wiktor et al., 2010) [171]	Other animals
*Strongyloides* spp.	67%	12	Microscopy	Mexico	(Mukul-Yerves et al., 2014) [172]	Other animals
*Strongyloides* spp.	57%	201	Microscopy	Estonia	(Oja et al., 2017) [173]	Other animals
*Strongyloides* spp.	47%	383	Microscopy	Mexico	(Ojeda-Robertos et al., 2017) [174]	Other animals
*Strongyloides* spp.	7%	6	Molecular	Iberian Peninsula	(Perera et al., 2013) [175]	Other animals
*Strongyloides* spp.	3%	468	Microscopy	Poland	(Pilarczyk et al., 2015) [176]	Other animals
*Strongyloides* spp.	17%	86	Microscopy	Bangladesh	(Rahman et al., 2018) [177]	Other animals
*Strongyloides* spp.	3%	1883	Microscopy	Italy	(Rinaldi et al., 2009) [178]	Other animals
*Strongyloides* spp.	44%	163	Microscopy	Portugal	(Rosalino et al., 2006) [179]	Other animals
*Strongyloides* spp.	45%	82	Microscopy	Australia	(Turni and Smales 2001) [180]	Other animals
*Strongyloides* spp.	UNK	UNK	Microscopy	Namibia	(Turner et al., 2010) [181]	Other animals
*Strongyloides* spp.	UNK	UNK	Microscopy	Namibia	(Turner et al., 2012) [182]	Other animals
*Strongyloides* spp.	<1%	213	Microscopy	Kenya	(VanderWaal et al., 2014) [183]	Other animals
*Strongyloides* spp.	74%	243	Microscopy	Philippines	(Ybanez et al., 2018) [184]	Other animals
